# Allergic diseases of the skin and drug allergies – 2024. Autoserum therapy in autologous serum skin test positive patients of chronic spontaneous urticaria

**DOI:** 10.1186/1939-4551-6-S1-P111

**Published:** 2013-04-23

**Authors:** Nidhi Sharma

**Affiliations:** 1 Department of Dermatology and Venerology, Dr D. Y Patil Medical College and Hospital, Mumbai, India

## Background

Chronic spontaneous urticaria (CSU) patients demonstrate functional autoantibodies against high affinity receptors of IgE (FcerI). Autologous serum skin test(ASST)is mostly used in chronic spontaneous urticaria to show its autoreactivity. ASST positive patients represent a severe group which can be benefited from autoserum therapy (AST). So, aim of our study is to evaluate the efficacy of autoserum therapy in ASST positive patients.

## Methods

AST was performed on 20 ASST positive patients (M:F = 12:8 ; age range = 19 - 45 years ; duration of the disease= 6months to 5 years). ASST was done by injecting patients own serum and normal saline as control in a dose of 0.05ml and readings were taken after 30 minutes. A wheal and flare response of more than 1.5mm diameter was considered as positive. ASST positive patients were given autoserum therapy in a dose of 0.05ml /kg intramuscularly once a week for 9 weeks. To evaluate the response we studied urticaria activity score (UAS), dermatology life quality index (DLQI) and disease severity by the no. of antihistamines used.

## Results

We found that out of 20 ASST positive patients 9 patients had significant response in terms of decrease UAS and antihistamines used with improvement in DLQI, 6 patients had moderate response and 5 patients had poor response.

## Conclusions

Autoserum therapy is effective in significant no. of ASST positive patients.

